# Aggregation-Induced Emission-Based Lateral Flow Immunoassay for Ultra-Sensitive and On-Site Detection of Porcine Epidemic Diarrhea Virus

**DOI:** 10.3390/bios15110736

**Published:** 2025-11-03

**Authors:** Bin Wang, Xufei Feng, Qian He, Hongwei Shi, Wei Hou, Jianjun Geng, Haidong Wang

**Affiliations:** 1 Department of Basic Veterinary Medicine, College of Veterinary Medicine, Shanxi Agricultural University, Jinzhong 030801, China; wangbin2022@sxau.edu.cn (B.W.); 20233874@stu.sxau.edu.cn (Q.H.); 20231061@stu.sxau.edu.cn (H.S.); weihou@sxau.edu.cn (W.H.); 2 Key Laboratory of Coal Environmental Pathogenicity and Prevention, Ministry of Education, School of Public Health, Shanxi Medical University, Taiyuan 030001, China; fengxufei@sxmu.edu.cn

**Keywords:** porcine epidemic diarrhea virus, aggregation-induced emission, lateral flow immunoassay, point-of-care testing, on-site detection

## Abstract

The porcine epidemic diarrhea virus (PEDV) has inflicted substantial economic losses on the swine industry, underscoring the need for sensitive point-of-care diagnostics. While lateral flow immunoassays (LFIA) offer rapidity and ease of use, traditional labels like colloidal gold suffer from limited sensitivity. Herein, we developed an aggregation-induced emission (AIE)-based LFIA, termed PED-ALFIA, for the highly sensitive detection of the PEDV antigen. PED-ALFIA exhibited a detection limit of 2.44 × 10^2^ TCID_50_/mL, which represents a 256-fold improvement in sensitivity over commercial colloidal gold kits and a 4-fold enhancement compared to our previously developed PED-TRFIA. The assay showed no cross-reactivity with other common swine viruses and demonstrated high reproducibility. When tested on clinical samples (n = 42), results showed 100% concordance with qPCR. Utilizing a portable fluorescence reader, the assay can be completed within 10 min, establishing PED-ALFIA as a sensitive, specific, and rapid on-site tool for the early diagnosis of PEDV.

## 1. Introduction

Porcine epidemic diarrhea virus (PEDV), an acute and highly contagious member of the Coronaviridae family that targets the intestinal tract, was first identified in the UK in the 1970s and has since achieved global distribution, posing a continuous threat to the swine industry [1]. PEDV infects pigs of all ages and breeds, causing clinical signs such as severe watery diarrhea, vomiting, dehydration, and anorexia [2]. It is particularly lethal in suckling piglets, with mortality rates often reaching 100%, leading to significant economic losses [3]. Recent continuous mutations have generated multiple PEDV genotypes and recombinant strains (e.g., GIIc) with enhanced pathogenicity and transmissibility, some capable of evading existing vaccine-induced immunity, thereby complicating control measures [4]. Since clinical symptoms closely resemble those of other porcine enteric coronaviruses, such as transmissible gastroenteritis virus (TGEV) [5] and porcine deltacoronavirus (PDCoV) [6], accurate differentiation based solely on clinical or pathological features is challenging. This underscores the critical need for a rapid, sensitive, and field-deployable detection method to enable early screening and timely intervention.

To facilitate the rapid and accurate detection of PEDV infection for effective control, various diagnostic technologies have been developed. These methods fall broadly into two categories: nucleic acid-based molecular techniques [7,8], such as polymerase chain reaction (PCR) [9], quantitative real-time PCR (qPCR) [10], cross priming amplification [11], recombinase polymerase amplification [12], and loop-mediated isothermal amplification [13]; and immunoassays that leverage high-affinity antigen–antibody interactions, including the indirect immunofluorescence assay (IFA) [14], enzyme-linked immunosorbent assay (ELISA) [15], and chemiluminescent immunoassay (CLIA) [16]. Compared to molecular methods, which often involve complex primer design, multi-step amplification, and tedious nucleic acid extraction, immunoassays generally feature simpler sample processing, easier operation, and superior storage stability, making them more suitable for rapid on-site screening. Furthermore, by directly targeting antigens or antibodies, immunoassays are particularly valuable for detecting early infections and conducting large-scale surveillance, offering additional advantages such as lower cost and easier implementation [17,18]. However, different immunoassays possess distinct strengths and limitations. IFA is highly sensitive, utilizing a fluorescently labeled secondary antibody to amplify signals for visual antigen localization. For instance, Liang et al. employed IFA to validate a monoclonal antibody against the PEDV S protein, achieving ultra-sensitive detection of recombinant S protein [14]. Despite its sensitivity, IFA is hampered by subjective interpretation, low throughput, poor suitability for automation, and susceptibility to photobleaching. ELISA, a high-throughput technique based on enzyme-conjugate antibodies and colorimetric substrate conversion, enables qualitative or quantitative analysis. Zhao et al. developed a double-antibody sandwich ELISA capable of detecting recombinant N protein at concentrations as low as 0.05 ng/mL [15]. While ELISA provides objective, quantifiable results, its sensitivity for very low analyte concentrations can be limited, and it involves multiple, time-consuming steps. CLIA, a more sensitive variant of ELISA, employs chemiluminescent substrates for ultra-trace detection via light emission. Tao et al. established a magnetic CLIA using antigen- or antibody-conjugate microspheres, which showed higher sensitivity and a reduced detection time of 30 min compared to conventional ELISA [16]. However, CLIA is constrained by unstable luminescence reactions and short signal duration, potentially affecting reproducibility. Notably, all these methods share common drawbacks, including complex procedures, dependence on specialized instrumentation, requirement for trained personnel, and the need for controlled laboratory environments, thus restricting their application primarily to centralized laboratories and hindering field deployment [19].

In recent years, developing point-of-care testing (POCT) compatible immunoassays has gained significant attention [20]. Core POCT attributes include rapidity, simplicity, portability, and minimal reliance on sophisticated equipment or technical expertise [21]. Emerging platforms such as microfluidic immunoassays [22], protein microarrays [23,24], and immunochromatographic tests have been introduced [25]. Among these, the LFIA based on paper substrates has emerged as one of the most successful POCT platforms due to its low cost, ease of use, rapid results, and hardware independence. Common LFIA labels include colloidal gold nanoparticles [26], colloidal carbon [27], and latex beads [28], which produce visible color bands upon aggregation. However, visual interpretation of color intensity, which is limited by the resolution of the human eye, restricts the sensitivity to semi-quantitative levels and can lead to false negatives at low target concentrations. To improve sensitivity and enable quantification, fluorescent labels such as FITC [29], quantum dots [30], and time-resolved fluorescent microspheres have been incorporated [31]. These materials exhibit high molar extinction coefficients and superior photostability, allowing instrumental detection of faint signals and improving sensitivity by 1-2 orders of magnitude. A persistent challenge, however, is aggregation-caused quenching (ACQ) [32], which limits the performance of conventional fluorophores at high labeling densities. Aggregation-induced emission fluorogens (AIEFs) [33], recognized as key nanomaterials for the future “nanophotonics revolution,” can overcome this limitation. Indeed, AIEgens have been successfully exploited in advanced diagnostic platforms for various targets, demonstrating exceptional performance owing to their aggregation-enhanced emission, high photostability, and superb signal-to-noise ratio [34,35]. However, their broader application in POCT faces persistent hurdles, including nonspecific adsorption in complex sample matrices, the scarcity of AIE probes with bright and stable near-infrared emission, and the challenge of translating laboratory-grade performance into robust, cost-effective, and user-friendly point-of-care devices [36,37]. Notably, despite these advances, the application of AIE technology for the detection of PEDV remains unexplored. In this work, we not only report the first AIE-based LFIA for PEDV but also tackle these general challenges directly by rationally designing a reliable AIE fluorescent microspheres (AIEFM) probe and meticulously optimizing the assay conditions, thereby developing a highly sensitive platform that bridges the gap between innovative material science and practical diagnostic needs.

**Figure 1:**
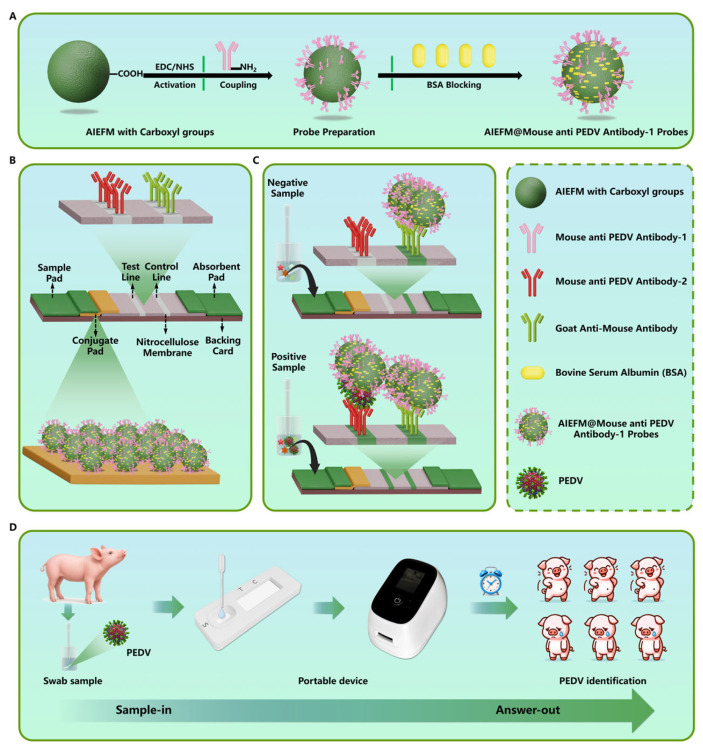
Schematic illustration of the PEDV detection using PED-ALFIA. (**A**) AIEFM@PEDV detection antibody probe preparation; (**B**) PED-ALFIA design; (**C**) PED-ALFIA mechanism; (**D**) PED-ALFIA application.

Based on this rationale, we developed an early on-site PEDV detection method using an AIE-based LFIA, designated PED-ALFIA (Figure 1). As illustrated in Figure 1A, AIEFM featuring surface carboxyl groups were covalently conjugate with PEDV detection antibodies to form the AIEFM@PEDV detection antibody probe. Figure 1B,C depict the architecture and principle of PED-ALFIA: the AIEFM@PEDV detection antibody probe is deposited on the conjugate pad, while PEDV capture antibody and goat anti-mouse IgG are immobilized on the T and C lines, respectively. Upon sample application, PEDV antigen binds to the AIEFM@PEDV detection antibody probe and migrates via capillary action. At the T line, a sandwich complex forms between the capture antibody and the AIEFM@PEDV detection antibody probe-antigen complex. Excess probe binds to the goat anti-mouse IgG at the C line. A fluorescence reader quantifies the fluorescence intensities of the T (FI_T_) and C (FI_C_) lines, and the FI_T_/FI_C_ ratio is used to determine the presence and concentration of PEDV. The reader, which measures 135 mm × 85 mm × 110 mm and weighs approximately 550 g, is powered by four AA batteries and can operate continuously for up to 10 h. As shown in Figure 1D, using a paper-based LFIA strip and a handheld fluorescence reader, PEDV antigen detection can be completed within 10 min via a simple workflow. In the following sections, we evaluate the performance and practical applicability of PED-ALFIA in terms of sensitivity, specificity, reproducibility, and accuracy, highlighting its potential for detecting other infectious diseases.

## 2. Materials and Methods

### 2.1. Materials

The PEDV GI strain, PEDV GII strain, pseudorabies virus (PRV), TGEV, and porcine reproductive and respiratory syndrome virus (PRRSV) were preserved at the Shanxi Key Laboratory for Prevention and Control of Major Animal Infectious Diseases (Jinzhong, China). Mouse anti-PEDV antibody-1 (Product Name: PEDV Detection Antibody, Catalog No.: JN110101, Host Species: Mouse), Mouse anti-PEDV antibody-2 (Product Name: PEDV Capture Antibody, Catalog No.: JN110102, Host Species: Mouse) and the PEDV Antigen Rapid Test Kit (Colloidal Gold), both targeting the PEDV nucleocapsid protein, along with the goat anti-mouse antibody (Cat#: JN2024712, host species: goat), were all purchased from Shanxi Jinnong Biotechnology Co., Ltd. (Jinzhong, China).PEDV, PRRSV, and PRV qPCR kits were obtained from Shanxi Jinnong Biotechnology Co., Ltd. (Jinzhong, China). and Hexu (Zhengzhou) Biotechnology Co., Ltd. (Zhengzhou, China). Fecal swab samples positive for PEDV were collected from pig farms in Shanxi Province. 1-Ethyl-3-(3-dimethylaminopropyl) carbodiimide hydrochloride (EDC), 2-Morpholinoethanesulphonic acid (MES), and N-hydroxysuccinimide (NHS) were purchased from Sigma-Aldrich (St. Louis, MO, USA). Surfactant S9 were purchased from Beijing Solarbio Science & Technology Co., Ltd. (Beijing, China). Bovine serum albumin (BSA) was obtained from Beyotime Biotechnology (Shanghai, China). Sodium hydroxide, sodium chloride, disodium hydrogen phosphate, sodium dihydrogen phosphate, potassium chloride, potassium dihydrogen phosphate, and methanol were supplied by Sinopharm Chemical Reagent Co., Ltd. (Shanghai, China). AIEFM were procured from the AIE Institute (Guangzhou, China). Goat anti-mouse antibody was purchased from Shandong Landu Biotechnology Co., Ltd. (Shandong, China). The fluorescence reader, capable of excitation at 360–390 nm and emission collection at 580–620 nm, was acquired from Helmen Precision Instruments Co., Ltd. (Suzhou, China). Nitrocellulose (NC) membranes, absorbent pads, and polyvinyl chloride (PVC) backing plates were sourced from Jinan Christie Bio-Technology Development Co., Ltd. (Jinan, China). Sample pads and conjugate pads were provided by Shanghai Jieyi Biological Technology Co., Ltd. (Shanghai, China). All data analysis was performed using OriginLab software (Version 2024; https://www.originlab.com/).

### 2.2. Preparation of AIEFM@PEDV Detection Antibody Probe

The AIEFM@PEDV detection antibody probe was prepared via covalent conjugation between carboxyl groups on AIEFM and amino groups of PEDV detection antibody. Briefly, 50 μL of 10 mg/mL AIEFM were washed twice with 5 mM MES buffer (pH 6.0) and resuspended to 2 mg/mL. Freshly prepared EDC and NHS solutions were added to final concentrations of 0.2 mg/mL and 0.6 mg/mL, respectively. The mixture was vortexed and incubated in the dark at room temperature with shaking for 30 min to activate carboxyl groups. After activation, the mixture was centrifuged at 8000× *g* for 10 min at room temperature, the supernatant was discarded, and the pellet was resuspended in 5 mM MES buffer (pH 6.5). 25 µg of PEDV detection antibody was added, and conjugation proceeded for 3 h at room temperature in the dark. The mixture was then centrifuged at 11,000× *g* for 20 min, the supernatant was removed, and the pellet was resuspended in a blocking solution (1% glycine, 0.05% BSA) and incubated for 1 h. After blocking, the probe was washed once with storage buffer (0.02 M Tris, 0.1% Tween-20, 0.5% BSA, 0.03% Proclin 300, pH 8.0) and finally resuspended in 200 µL of the same buffer to a final concentration of 2.5 mg/mL for storage at 4 °C.

### 2.3. Characterization of AIEFM@PEDV Detection Antibody Probe

The storage stability of AIEFMs was assessed by visual inspection under UV light after storage at 4 °C for 28 days. Morphology and microstructure were characterized by scanning electron microscopy (SEM, ZEISS GeminiSEM 500, Oberkochen, Germany) and transmission electron microscopy (TEM, JEM-2100F, JEOL, Akishima, Japan), respectively. Elemental analysis was performed using an energy-dispersive X-ray spectrometer (EDS) attached to the TEM. Hydrodynamic diameter was measured by dynamic light scattering (DLS, Nano-Zetasizer ZS90, Malvern Panalytical, Malvern UK). Fluorescence spectra and photostability of AIEFM and the AIEFM@PEDV probe were characterized using a microplate reader (Infinite M Plex, Tecan, Männedorf, Switzerland). For photostability, AIEFMs were continuously exposed to UV light in a biosafety cabinet for 20 min, and fluorescence intensity was monitored.

### 2.4. Fabrication of PED-ALFIA Test Strips

Test strips comprised a sample pad, conjugate pad, NC membrane, and absorbent pad assembled sequentially on a PVC backing sheet. The sample pad was soaked in 10 mM PBS (pH 7.4) containing 1% BSA and 0.5% Tween-20, then dried overnight at 45 °C. The conjugate pad was treated with 50 mM Tris (pH 8.0) containing 5% sucrose, 3% trehalose, and 1% BSA, and dried overnight at 45 °C. The AIEFM@PEDV probe, diluted 6-fold, was uniformly sprayed onto the conjugate pad using a 3D dispensing system and dried at 37 °C for 8 h. On the NC membrane, the PEDV capture antibody (0.5 mg/mL, T line) and goat anti-mouse IgG (1.0 mg/mL, C line) were dispensed at 1 µL/cm with a 5 mm gap between lines and dried at 37 °C for 6 h. All components were assembled on the PVC backing with 1–2 mm overlaps, cut into 3.9 mm wide strips, and mounted into plastic cartridges.

### 2.5. Establishment of the PED-ALFIA Method

A PEDV positive control with a titer of 10^7^ TCID_50_/mL was serially diluted. Key parameters including the amount of detection antibody conjugate, the concentration of capture antibody on the T line, the composition of the sample dilution buffer, and the immunoreaction time were optimized.

The conjugation amount of PEDV detection antibody (10, 25, 50, 75, 100 µg) was optimized based on the highest signal-to-noise ratio between T-line intensities of positive and negative samples. T-line coating concentrations of PEDV capture antibody (0.1, 0.25, 0.5, 0.75, 1.0 mg/mL) were tested, selecting the concentration where the positive signal plateaued without increasing the negative signal. Three dilution buffers were evaluated: Buffer A (10 mM PBS with S9 and Proclin, pH 8.0), Buffer B (50 mM Tris with S9 and Proclin, pH 8.0), Buffer C (50 mM Tris with S9, Proclin, and NaCl, pH 8.0). The optimal buffer was selected based on the highest FI_T_/FI_C_ ratio for negative samples and positive samples diluted 1280- and 40-fold. Immunoreaction time (5–30 min) was optimized by identifying the time point where the positive signal plateaued.

The optimized procedure was as follows: 10 µL of sample was mixed with 90 µL of dilution buffer (Buffer C). Then, 80 µL of the mixture was added to the sample well. After 10 min, a portable fluorescence reader quantified the FI_T_ and FI_C_, and the FI_T_/FI_C_ ratio was calculated. A sample was considered positive if its FI_T_/FI_C_ value was ≥3 times that of the negative control.

### 2.6. Evaluation of PED-ALFIA Performance

Sensitivity was determined by testing serial dilutions of the PEDV positive control in triplicate. The visual limit of detection (LOD) was determined under UV light. The analytical LOD was defined as the concentration yielding a T/C signal-to-noise ratio ≥3. Specificity was assessed by testing PRV, PRRSV, TGEV, PEDV GI, and PEDV GII strains; cross-reactivity was evaluated using FI_T_/FI_C_ values. Repeatability was determined by testing positive and negative reference samples 15 times independently; the coefficient of variation (CV) of FI_T_/FI_C_ values was calculated. Clinical utility was assessed using 42 porcine fecal swab samples. For the fecal swab samples, 100 μL of PBS buffer was added to each tube. The tubes were tightly capped and vigorously vortexed to ensure complete dissolution of the fecal material. Then, 10 μL of the supernatant was collected as the test sample and analyzed using the standard PED-ALFIA procedure. The results were compared with those from a commercial qPCR kit, and the concordance rate was analyzed. All experiments were performed in triplicate.

## 3. Results

### 3.1. Characterization of AIEFM and AIEFM@PEDV Detection Antibody Probe

**Figure 2:**
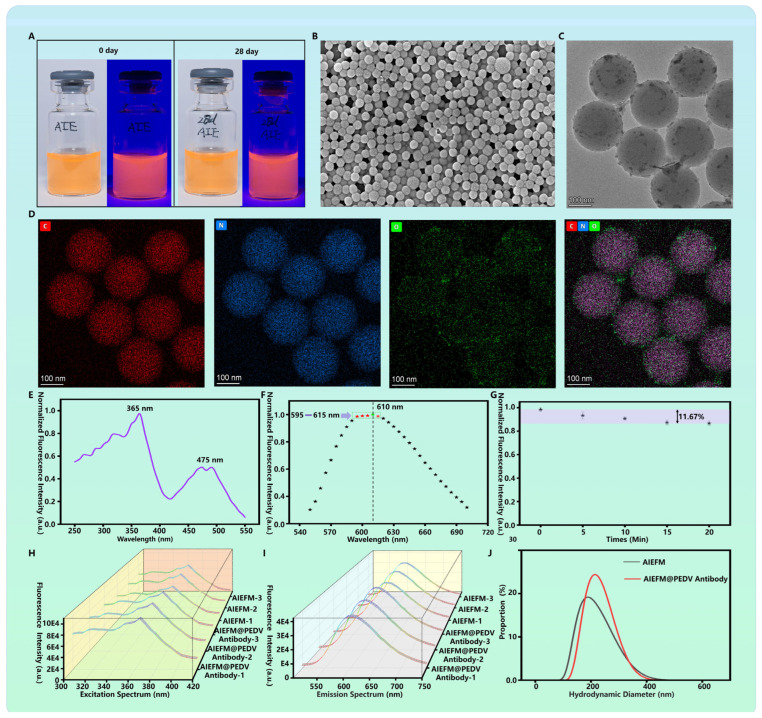
AIEFM characterizations. (**A**) Freshly prepared AIEFM and AIEFM stored for 28 days under UV illumination; (**B**) SEM image of AIEFM; (**C**) TEM image of AIEFM; (**D**) TEM-EDS elemental mapping of AIEFM; (**E**) Excitation spectrum of AIEFM; (**F**) Emission spectrum of AIEFM; (**G**) Fluorescence intensity of AIEFM after various durations of UV irradiation; (**H**) Excitation spectra of unconjugated AIEFM and AIEFM@PEDV detection antibody probes; (**I**) Emission spectra of unconjugated AIEFMs and AIEFM@PEDV detection antibody probes; (**J**) Hydrodynamic diameter distributions of AIEFM and AIEFM@PEDV detection antibody probes.

The successful preparation and stability of AIEFM and AIEFM@PEDV Detection Antibody Probe are fundamental to the assay’s performance. As shown in Figure 2A, both freshly prepared AIEFM and those stored at 4 °C for 28 days exhibited a homogeneous dispersion without visible aggregation or sedimentation. Under UV light illumination, both samples emitted bright red fluorescence, indicating excellent storage stability of the AIEFM. Morphological characterization using scanning electron microscopy (SEM) revealed that the AIEFM were uniformly spherical with a smooth surface and an average diameter of approximately 190 nm (Figure 2B). Further examination by transmission electron microscopy (TEM) confirmed the spherical morphology and indicated a high specific surface area, which is advantageous for conjugating a large number of antibody molecules (Figure 2C). Elemental analysis via energy-dispersive X-ray spectroscopy (EDS) confirmed that the microspheres were primarily composed of carbon and oxygen, consistent with their carboxylated polymer composition (Figure 2D). The optical properties were critically evaluated. The fluorescence excitation spectrum of the AIEFM, measured with a microplate reader, showed an optimal excitation wavelength at 365 nm (Figure 2E). The emission spectrum displayed a major peak at 610 nm, with additional peaks of similar intensity between 595 and 615 nm (Figure 2F). To evaluate photostability, the AIEFM were continuously exposed to UV light in a biosafety cabinet for 20 min. The fluorescence intensity decreased by only 11.67%, demonstrating strong resistance to photobleaching (Figure 2G). The conjugation of PEDV detection antibodies to the AIEFM was confirmed through spectral analysis and dynamic light scattering (DLS). The excitation and emission profiles of the resulting AIEFM@PEDV detection antibody probe remained consistent with those of the bare AIEFM (Figure 2H,I), indicating that the conjugation process did not alter the core optical properties of the fluorogen. Most importantly, DLS measurements showed a significant increase in the hydrodynamic diameter from 194.9 nm for the bare AIEFMs to 221.1 nm for the AIEFM@PEDV detection antibody probe (Figure 2J), providing clear evidence of successful antibody conjugation.

### 3.2. Optimization of PED-ALFIA

**Figure 3:**
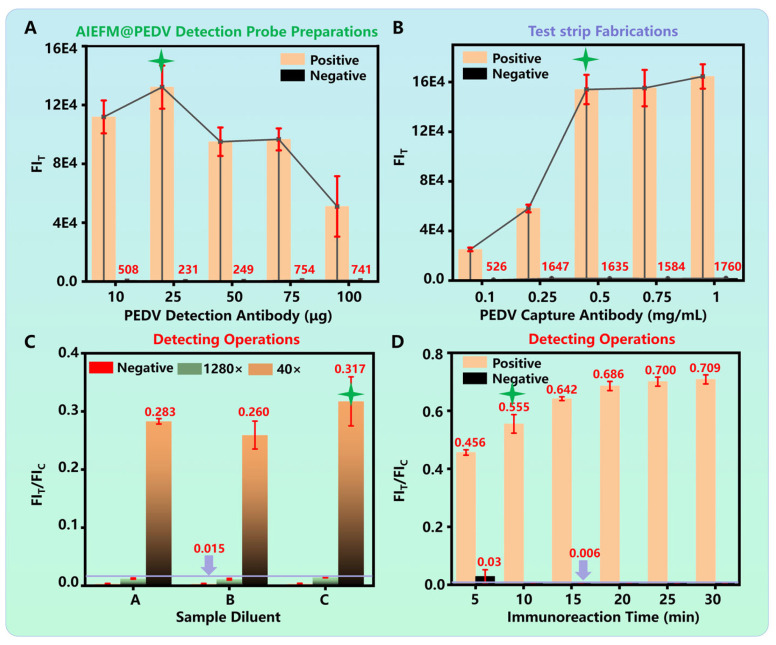
Optimization of key parameters for PED-ALFIA. (**A**) Optimization of the coupling amount of PEDV detection antibody. (**B**) Optimization of the concentration of PEDV capture antibody. (**C**) Evaluation of different dilution buffers. (**D**) Optimization of immunoreaction time. (The green symbol in the figure represents the optimal condition).

To achieve the best detection performance, we systematically optimized the key parameters of the PED-ALFIA. First, the amount of PEDV detection antibody conjugate to a fixed quantity of AIEFM was optimized. We tested amounts of 10, 25, 50, 75, and 100 μg. The results indicated that conjugating 25 μg of antibody yielded the maximum FI_T_ for positive samples while maintaining a minimal background signal for negative samples (Figure 3A). This amount was therefore selected as the optimal conjugation amount.

Next, the concentration of the PEDV capture antibody dispensed on the T line was optimized. A gradient of concentrations (0.1, 0.25, 0.5, 0.75, and 1.0 mg/mL) was tested. As shown in Figure 3B, the FI_T_ signal for positive samples increased with the capture of antibody concentration and reached a plateau at 0.5 mg/mL. The signal for negative samples remained low across all concentrations. Thus, 0.5 mg/mL was chosen as the optimal capture antibody concentration.

The composition of the sample dilution buffer was also found to be critical for efficient release of the probe from the conjugate pad and for minimizing non-specific binding. Among the three buffers tested (Buffer A: PBS-based; Buffer B: Tris-based; Buffer C: Tris-based with NaCl), Buffer C yielded the highest FI_T_/FI_C_ ratios for positive samples (diluted 1280-fold and 40-fold) while keeping the FI_T_/FI_C_ ratio for the negative control very low (Figure 3C). This demonstrated that Buffer C provided the best assay dynamics and was selected for all subsequent experiments.

Finally, the immunoreaction time was optimized. The FI_T_/FI_C_ ratio for positive samples increased over time and stabilized after 10 min, whereas the ratio for negative samples remained consistently low (Figure 3D). Consequently, a 10-min reaction time was established as the standard for the PED-ALFIA protocol, offering a perfect balance between speed and sensitivity.

### 3.3. Analytical Performance and Clinical Validation of PED-ALFIA

**Figure 4:**
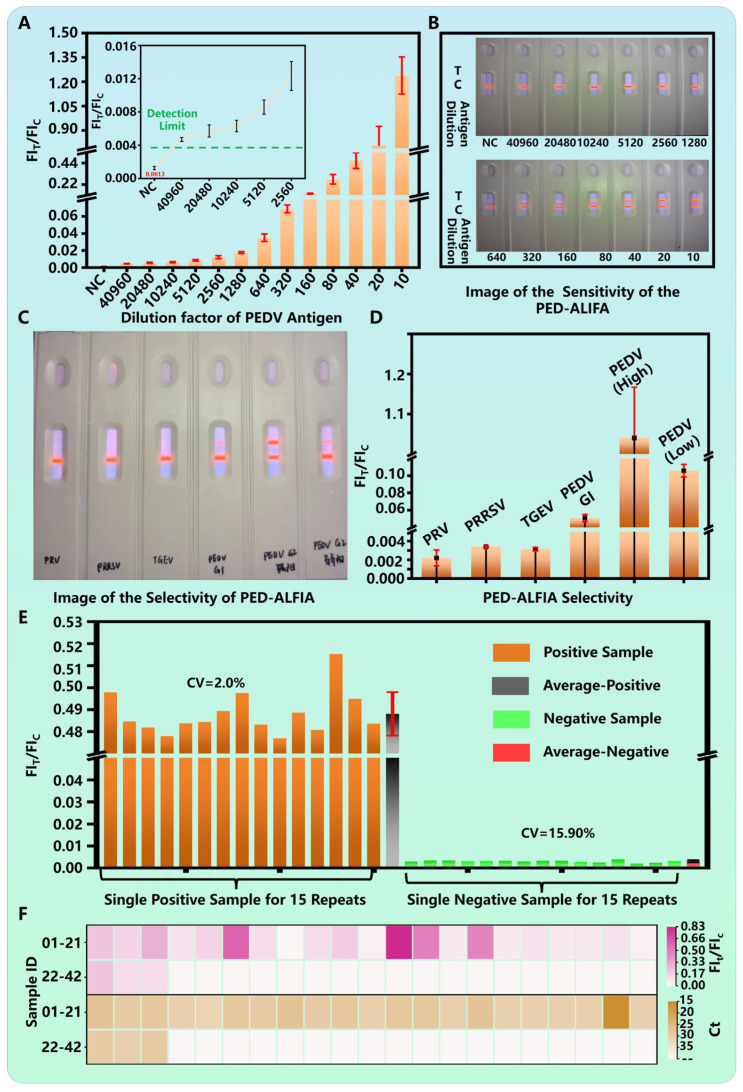
PED-ALFIA performances. (**A**) FI_T_/FI_C_ ratios of PEDV positive standard samples with varying dilution factors; (**B**) Photographs of PED-ALFIA of detecting PEDV; (**C**) photographs and (**D**) results of detecting PRRSV, PRV, TGEV, PEDV GI and PEDV GII samples; (**E**) FI_T_/FI_C_ ratios of PEDV positive and negative samples for 15 repeated detections. (**F**) Analysis of PED-ALFIA detection using real clinical samples.

The sensitivity of the optimized PED-ALFIA was rigorously evaluated. A two-fold serial dilution series of a PEDV positive standard (initial titer: 10^7^ TCID_50_/mL) was tested. The FI_T_/FI_C_ ratio showed a concentration-dependent increase (Figure 4A). The limit of detection (LOD) was defined as the lowest concentration that produced a FI_T_/FI_C_ signal at least three times greater than that of the negative control. Based on this criterion, the analytical LOD was determined to be at a dilution of 1:40,960, corresponding to a viral titer of 2.44 × 10^2^ TCID_50_/mL (Figure 4A, inset). Under UV light, a distinct red test line was visibly clear at this LOD dilution, confirming the high visual detection sensitivity (Figure 4B). This sensitivity represents a 4-fold improvement over our previously developed PED-TRFIA [31] and a remarkable 256-fold enhancement compared to commercial colloidal gold-based LFIA kits [31].

The specificity of PED-ALFIA was assessed by testing it against other common swine viruses, including PRV (5 × 10^4^ TCID_50_/mL), PRRSV (5 × 10^4^ TCID_50_/mL), and TGEV (5 × 10^4^ TCID_50_/mL). The results showed that the FI_T_/FI_C_ ratios for these non-target viruses were all below the positive threshold and indistinguishable from the negative control (Figure 4C,D), indicating no cross-reactivity. Importantly, the assay effectively detected both PEDV genotype GI and GII strains, confirming its broad reactivity against currently circulating variants.

The reproducibility of the assay was excellent. Fifteen independent replicate tests were performed using a PEDV-positive standard and a negative control. The coefficients of variation (CV) for the FI_T_/FI_C_ values were calculated to be 2.0% for the positive sample and 15.9% for the negative sample (Figure 4E), demonstrating high repeatability and reliability.

To validate the clinical utility, 42 clinical fecal swab samples comprising 24 qPCR-confirmed positive cases and 18 negative cases were analyzed using PED-ALFIA. The results demonstrated 100% concordance with the qPCR findings, achieving perfect agreement in all tested samples (Figure 4F). This high level of consistency within complex clinical matrices indicates the promising clinical utility of PED-ALFIA for on-site diagnosis.

## 4. Discussion and Conclusions

PEDV is a highly pathogenic coronavirus that can infect pigs at all growth stages, and no commercial antiviral drugs are currently available. Studies have shown that early intervention is crucial for effective control of PEDV infection, underscoring the need for a simple, rapid, and highly sensitive on-site detection method. As shown in Table 1, various detection methods for PEDV have been compared. Currently, qPCR is regarded as a gold standard for PEDV nucleic acid detection due to its high sensitivity and specificity. However, its dependence on bulky instruments and complex nucleic acid extraction procedures limits its field applicability. ELISA, commonly used for antigen detection, is also unsuitable for on-site use because it requires a microplate reader and involves multiple washing steps. Although traditional LFIAs allow field testing, their key limitation lies in sensitivity. Common strategies to enhance LFIA sensitivity include: (1) improving the signal probe, (2) optimizing the biochemical reaction, and (3) refining test strip materials. Since commercially available test strips generally use standardized components, such as NC membranes, absorbent pads, and PVC backing plates, we focused on optimizing the signal probe and the biochemical reaction parameters. For PEDV detection, we systematically optimized key experimental conditions, including the coupling amount of the detection antibody, the concentration of the capture antibody, the composition of dilution buffers, and immunoreaction time (see Figure 3 for details). These optimizations contributed to a high-performance PEDV detection system, particularly under clinical conditions (see Figure 4). Beyond biochemical optimization, we also improved the signal probe, which significantly enhanced detection sensitivity (see Table 1). Compared to conventional colloidal gold nanoparticles and time-resolved fluorescent microspheres, our design achieved markedly higher sensitivity.

In this study, we employed AIEFM as the signal label. Unlike conventional fluorescent materials, AIEFMs exhibit enhanced emission upon aggregation, which helps avoid ACQ and facilitates the development of a highly sensitive PED-ALFIA. In a comprehensive evaluation, PED-ALFIA demonstrated outstanding performance: the LOD reached a dilution of 1:40,960 (equivalent to 2.44 × 10^2^ TCID_50_/mL), representing a 4-fold improvement over a previously developed PED-TRFIA and a 256-fold increase in sensitivity compared to commercial colloidal gold strips. The assay showed high specificity, with no cross-reactivity against common swine pathogens such as PRV, PRRSV, and TGEV, and it reliably detected both PEDV genotypes GI and GII. Reproducibility was excellent, with CVof 2.0% for positive samples and 15.9% for negative samples. When testing 42 clinical fecal samples, PED-ALFIA results showed 100% concordance with qPCR. In summary, PED-ALFIA is a sensitive, specific, reproducible, and clinically accurate method suitable for rapid on-site screening and monitoring of PEDV, holding strong potential for field applications such as farm-based testing.

## Figures and Tables

**Table 1:** Comparisons of the methods designed for PEDV detection.

Detection Techniques	Cost	Instrument	LOD	Assay Time	On-site Detection	Reference
qPCR	High	PCR Instrument	7.5 × 10^2^ RNA Copies/Reaction	>1 h	NO	[10]
ELISA	High	Microplate Reader	0.05 ng/mL	>1 h	NO	[15]
Conventional Gold-LFIA	Cost-ffective	NO	6.25 × 10^4^ TCID_50_/mL	<15 min	YES	[31]
PED-TRFIA	Cost-effective	Fluorescence Reader	9.76 × 10^2^ TCID_50_/mL	<15 min	YES	[31]
PED-ALFIA	Cost-effective	Fluorescence Reader	2.44 × 10^2^ TCID_50_/mL	<15 min	YES	This Work

## Data Availability

The data supporting this study’s findings are available from the corresponding author upon reasonable request.

## Abbreviations

The following abbreviations are used in this manuscript:

PEDV	Porcine epidemic diarrhea virus
TGEV	Transmissible gastroenteritis virus
LFIA	Lateral flow immunoassays
AIE	Aggregation-induced emission
PDCoV	Porcine deltacoronavirus
PCR	Polymerase Chain Reaction
qPCR	Quantitative real-time PCR
ELISA	Enzyme-linked immunosorbent assay
CLIA	Chemiluminescent immunoassay
POCT	Point-of-care testing
ACQ	Aggregation-caused quenching
AIEFM	AIE fluorescent microspheres
PRV	Pseudorabies virus
PRRSV	Porcine reproductive and respiratory syndrome virus
DLS	Dynamic light scattering
EDS	Energy-dispersive X-ray spectroscopy
LOD	Limit of detection
CV	Coefficients of variation
